# *“The needs have clearly evolved as time has gone on*.”: A qualitative study to explore stakeholders’ perspectives on the health needs of Syrian refugees in Greece following the 2016 European Union-Turkey agreement

**DOI:** 10.1186/s13031-018-0158-9

**Published:** 2018-06-08

**Authors:** Rebecca Hémono, Bridget Relyea, Jennifer Scott, Sinan Khaddaj, Angeliki Douka, Alison Wringe

**Affiliations:** 1 Women and Health Alliance International, 160 bis Rue du Temple, Paris, France; 2 École des Hautes Études en Santé Publique, 15 Avenue du Professeur Léon Bernard, Rennes, France; 3 Women and Health Alliance Hellas, Athens, Greece; 4 Beth Israel Deaconess Medical Center, Department of Obstetrics and Gynecology, 330 Brookline Avenue, Boston, MA USA; 5 Harvard Medical School, Department of Obstetrics, Gynecology and Reproductive Biology, 25 Shattuck Street, Boston, MA USA; 6 London School of Hygiene and Tropical Medicine, Department of Population Health, Keppel Street, London, UK

**Keywords:** Refugee, Migration, Health needs, Syria, Greece, Europe, Qualitative

## Abstract

**Background:**

By 2017, more than 500,000 Syrian refugees had passed through Greece seeking safety and asylum. Understanding how their health needs evolved over the refugee crisis in Greece and in relation to changing migration policy, and exploring the challenges involved in delivering their healthcare is timely as non-governmental organizations (NGOs) transition health service provision to the Greek health authorities.

**Methods:**

We conducted a qualitative study to explore stakeholders’ perspectives on delivering health services to Syrian refugees over the course of the humanitarian response in Greece from 2015 to 2017. Twenty-five in-depth interviews were conducted face-to-face or by Skype with healthcare providers, NGO staff, and organizational and government representatives involved in coordinating and managing healthcare for the Syrian refugee population in Greece. Following informed consent, interviews were audio-recorded or detailed summaries were manually recorded. Data were coded inductively to identify emerging themes.

**Results:**

Following the implementation of the European Union-Turkey agreement in 2016, healthcare providers in refugee camps reported a shift from acute physical health issues to mental health disorders, and heightened risks of gender-based violence among Syrian refugees. Key challenges to service delivery included a narrow model of healthcare provision and insufficient referral mechanisms for social support and mental health services. Language and gender differences between refugees and healthcare providers, and a lack of privacy and space in clinics impeded the quality of care. Stakeholders observed deterioration in refugees’ mental health in relation to longer periods spent in the camps. Many also emphasized that services for gender-based violence and mental health should be prioritized.

**Conclusion:**

This study provides stakeholders’ perspectives on changes in refugee health needs over the course of the humanitarian response in Greece. With protracted encampment resulting from migration policy, addressing mental health disorders and gender-based violence should be prioritized, including psychosocial training for healthcare providers and strengthening referral mechanisms for specialized care. The findings also emphasize the importance of providing human-centered care and gender concordant services by incorporating female healthcare providers and interpreters into medical teams. Strategic communication and coordination is needed between NGOs and Greek health authorities to facilitate the transition of health service delivery to the Greek healthcare system and to improve access and quality of care for refugees.

## Background

The civil war and protracted crisis in Syria have led to widespread displacement [1], with nearly five million people seeking refuge in neighboring countries of Jordan, Lebanon, Iraq, Egypt, and Turkey, and the Eastern Mediterranean and Western Balkans region since 2011 [2–4]. In 2015, more than 450,000 Syrian refugees crossed the Mediterranean Sea to Europe [5]. Most refugees from Syria did not intend to remain in Greece and were seeking asylum and family reunification in other European Union (EU) Member States [6]. They were typically transferred to the Greek mainland within one to three days before continuing on the Western Balkan transit route. Migration policy changed in late 2015 when screening and registration processes were implemented in Greece, and Macedonia and other Balkan countries closed their borders, effectively creating a bottleneck of refugees in northern Greece.

In March 2016, an agreement between the EU and Turkey, known as the EU-Turkey Statement, came into effect, which called for all “irregular” migrants crossing into Greece to be returned to Turkey, as well as a commitment from Turkey to restrict new sea or land migration routes from Turkey to the EU [7–9]. This agreement led to a diminished influx of refugees into Greece [10]. Refugee outflows from Greece also decreased as a result of border closures to its East, leaving approximately 60,000 individuals, predominantly from Syria, stranded on the Greek mainland and islands waiting for asylum claims to be processed [10, 11].

The influx of refugees into Greece in 2015 prompted an international response to support the government and local authorities in providing humanitarian assistance, including medical relief aid. Numerous national and international non-governmental organizations (NGOs) were mobilized to provide health services, with coordination and oversight provided by the United Nations High Commissioner for Refugees (UNHCR) [10]. By the middle of 2017, in the context of fewer new arrivals and reduced funding from donors and international organizations, many NGOs began the process of transitioning health service provision to the local health authorities [12].

Reports from UNHCR documented more than 45,500 refugees and migrants in Greece as of September 2017 [13], however there has been a paucity of empirical research documenting their health needs, including how these needs have evolved in the context of the border closures and migration policy changes, and the challenges in delivering services to meet these needs. Studies on Syrian refugee health needs outside of Europe, including in camp settings in Lebanon, have indicated a high burden of non-communicable diseases (NCDs) [14, 15], risks of gender-based violence (GBV) [16], and insufficient access to sexual and reproductive healthcare [17], but the extent to which these findings reflect the situation in Europe is unclear. Some assessments in Europe have pointed to psychological risks among refugees related to war, violence and trauma [18, 19], protection needs among women and girl refugees [20], challenges in managing mental health disorders [21] and NCDs [22, 23]. However, few studies have explored how these health needs evolved over the course of the refugee crisis in Greece and in relation to policy changes, nor considered the perspectives of healthcare providers and government representatives from the host country managing and coordinating healthcare. This limits our understanding of how effective the humanitarian response has been in meeting Syrian refugees’ health needs, and what lessons can be learned for the future delivery of health services for this population.

This study aimed to understand stakeholders’ perspectives on the health needs of Syrian refugees in Greece and how these perspectives and health needs evolved in relation to changing migration policy during the course of the humanitarian response in Greece (2015 to 2017). The study also aimed to document the challenges that stakeholders experienced in responding to these needs, in order to improve the quality of care for the refugee population in the future.

## Methods

This qualitative study was designed to assess the period of the humanitarian response in Greece from the acute phase of the refugee crisis beginning in 2015, to the transition of health service delivery from NGOs to Greek health authorities in 2017. Data collection was conducted from June 2016–June 2017.

### Study setting

The study was conducted in open and closed refugee camps and alternative accommodation facilities (AAFs) on five Greek islands (Kos, Chios, Samos, Leros and Lesbos) and in Thessaloniki in northern Greece on the border of Macedonia. Study locations were selected based on where NGOs were involved in the delivery of health services to Syrian refugees. These locations hosted refugees predominantly from Syria, Afghanistan, Iraq, and Pakistan; however, the data collection focused on the needs of Syrian refugees.

The refugee camps were either classified as “open” or “closed”, hosting between approximately 100 to 1500 refugees. On the islands, open camps were located on the waterfront, with accommodation typically comprising tents made from plastic sheeting. Closed camps were generally situated away from towns, with accommodation being provided in rows of metallic transport containers, and with shared communal washing and cooking areas. In some instances, these closed camps were situated in gated, former military barracks with limited opportunities for refugees to leave. Alternative accommodation facilities typically included hotel rooms in city centers or housing complexes and apartments on the outskirts of towns, and were designated for individuals deemed by UNHCR to be vulnerable to risks of violence or poor health outcomes, and pregnant women.

Health services available to the refugees in the camps and AAFs included those provided by NGOs in temporary clinics located within the camps. Medical care was also provided by NGOs through household visits for residents in AAFs. Specialized care was mostly provided by the Greek health authorities through referrals made from NGOs to larger health facilities and hospitals.

### Participant sampling

Purposive and snowball sampling methods were used to recruit participants with a range of experiences in the delivery, management or coordination of health services for Syrian refugees in Greece. This included healthcare providers from national and international NGOs, staff from national and international NGOs, and organizational and government representatives involved in coordinating and managing healthcare. Potential participants were contacted through a partner organization, Women and Health Alliance International (WAHA), an international NGO providing healthcare services in refugee camps and AAFs. Purposive sampling was used to sample healthcare providers and stakeholders that had been involved in the humanitarian response since the onset of the crisis in Greece and over the course of the response. Subsequently, snowball sampling was used to access a more diverse range of stakeholders and healthcare providers.

### Data collection

Data were collected through in-depth interviews administered by three interviewers.

Interviewers were trained in the ethics of conducting qualitative health research and interviewing techniques, and were not previously known to the participants. Written information sheets outlining the study were provided to the participants, and interviews were conducted after written informed consent was obtained. Interview questions explored participants’ perceptions of Syrian refugees’ health needs, including mental health and sexual and reproductive health needs, participants’ views on how Syrian refugee health needs had evolved over time and in relation to changes in migration policy, health service provision and referral pathways, as well as barriers and facilitating factors to delivering healthcare or managing the provision of health services. Interviewers emphasized that the study’s focus of interest was participants’ perceptions of health needs and experiences with Syrian refugees*.* Interviews were conducted over Skype or in person in Greece in English, French or Greek at the choice of the participant, and lasted approximately 60 min. The interviews were audio-recorded, or where consent was only provided to take notes, detailed summaries of the interview were manually recorded. De-identified audio files were transferred to a secure server, accessible only by the research staff, and deleted from recording devices.

### Analysis

Following each interview, the interviewer produced field notes and reviewed emergent themes. Interviews were either transcribed, and if conducted in Greek or French, were translated into English, or detailed interview summaries were prepared. All identifying information was removed from interview transcripts. Data analysis was undertaken using a thematic approach [24]. The transcripts and interview summaries were coded manually both inductively and iteratively to identify emerging ideas and concepts that were grounded in the participants’ accounts. Detailed analytic memos were produced and the emerging concepts were then distilled into themes and incorporated within a coding framework that was reviewed, discussed and refined by the co-authors before being applied to the remaining transcripts. The coded data were then reviewed to explore emerging patterns and meanings in relation to the study aims and objectives.

### Ethics

Human subjects research approval was obtained from the London School of Hygiene and Tropical Medicine and the Hellenic Data Protection Authority in Greece.

## Results

In total, 31 individuals were invited to participate in the study, of whom six refused due to not having explicit organizational approval to participate, or due to a lack of time. The characteristics of the 25 individuals who participated in the study are shown in Table 1.

**Table 1:** Demographic characteristics of study participants

	Male (N = 7)	Female (N = 18)	Total (N = 25)
Respondent category			
Healthcare Provider	2	6	8
Social Worker	1	4	5
National NGO Staff	1	3	4
International NGO (INGO) Staff	3	5	8
Location of service provision			
Greek Islands (Chios, Samos, Kos, Leros, Lesbos)	6	14	20
Greek Mainland	1	4	5

Three key themes emerged from the analysis which reflected the evolving nature of the refugees’ health needs and the corresponding challenges in health service provision.

### Changes in migration policy and implications for refugee health

Most stakeholders described how healthcare needs of the refugee population evolved over time and during the course of policy changes and implementation. Before the EU-Turkey agreement was implemented, medical care in NGO clinics was largely limited to consultations for acute infections, hypothermia and wounds, provision of over-the-counter pain medication and antibiotics for individuals that had recently arrived by sea. As one healthcare provider on the islands explained:

*Some [refugees] just came from a really long journey on the sea so they were wet. Some of them crossed during the day…there were many cases of sunburn, dehydration...many of them had blisters, scars…we were curing many of these types of things and then [providing] primary healthcare…At that time, September, October, November 2015, when people got out of the boat…people that were not properly injured were not looking to stay a long time with us. They were just passing through for 15, 30 min, a few hours and then going and walking…to cross to the mainland and the Balkan route… they were not interested in staying with us. (Healthcare provider, Greek islands).*

While most healthcare providers felt the provision of acute care during the emergency phase of the crisis was appropriate, they reported that chronic diseases were not being systematically screened for, and that adequate secondary care was often not sought or was not readily accessible. Many participants reported difficulties in addressing chronic disease due to the ongoing movement of refugees across borders, and the challenges of providing care for refugees who had arrived without previous personal health records or prescriptions. Healthcare providers also reported that prior to border closures and to the EU-Turkey agreement, almost all refugees intended to continue along the Western Balkan migrant route and were reluctant to take up local referrals in Greece, for fear that it would delay their passage. The provision of care was therefore limited to treating urgent and acute needs and providing first-aid to refugees arriving by sea, despite some refugees’ needs for longer-term care.

Following the change in migration policies, the refugees’ movements became restricted and much of the refugee population transitioned into formal camps. Healthcare providers observed a shift in health-seeking behaviors after the population moved into camps. They reported that those with chronic conditions were more likely to seek out care, regardless of whether they had medical records or former prescriptions. They also described how this transition substantially shifted the nature of the consultations being provided in clinics:

*“People were coming from Turkey and staying 72 hours maximum in Chios and then going on to other islands... after a while, the population started to be stuck and now we have people who have been living here for five months. So, the process and the medical treatment are certainly not the same... we [went] from [an] emergency program to long-term care and it’s certainly different... the needs have clearly evolved as time has gone on.” (Healthcare provider, Greek islands)*

Healthcare providers noted that some refugees came to camp clinics for re-diagnosis and confirmation of treatments for conditions that had been managed in Syria, but not reassessed since their departure. Cases that required specialized care were generally referred, however stakeholders noted gaps in referral systems and care, or that medications that were not systematically stocked by the NGOs or local pharmacies; thus, limiting the scope of services that could effectively be provided by NGOs and leaving some unmet health needs.

### Addressing emerging psychosocial needs in the closed-border context

As refugees stayed for prolonged periods in the camps, many stakeholders observed an increasing frequency of mental health disorders, including symptoms of depression, anxiety and post-traumatic stress disorder among some refugees. Stakeholders reported an association between the lengthy and uncertain asylum process and poor living conditions and the decline in mental health among refugees. One healthcare provider related these symptoms to the uncertainties refugees faced:

*[There is] no project, no target, no purpose. Nothing, just waiting…Just waiting for nothing. And it’s mysterious. Nobody knows [their] future. They are very [pre]occupied…Panic! (Healthcare provider, Greek mainland)*

Stakeholders described a parallel increase in the demand for psychosocial support among refugees in the context of policy change and protracted encampment. Healthcare providers reported that within the camps, they were able to build trust among beneficiaries, and described refugees coming regularly to health clinics to discuss their psychosocial problems. Some healthcare providers felt able to provide the type of care that refugees were seeking during consultations, however it was more challenging for others:

*I need ten hours [a day] because each patient [tells] me [their] story for a long time. And I think this is the most important part of my mission… I try to explain that to my nurse… I know it will make her exhausted because our job is long, but I think this is very important part. To listen, to support them, to encourage them. (Healthcare provider, Greek islands)*

Several stakeholders also described the rise in mental health disorders alongside an increase of violence within the camps. Prior to the EU-Turkey agreement, healthcare and social work providers reported that they observed risks of GBV for refugees in transit. However, they often felt limited in their ability to provide adequate GBV response services due to short consultation visits and insufficient privacy and space within the health clinics to address sensitive needs. In later interviews, stakeholders noted that the risks of GBV for refugees had increased over time, and were particularly prominent in camps with large populations of single men, for women traveling alone and for minors. The presence of illicit substances and the rise of substance use disorders among refugees were also seen by many stakeholders to exacerbate existing risks of violence.

*It is not a camp, it’s just a jungle. (Social worker, Greek mainland)*

Healthcare providers acknowledged the need for targeted care and referral mechanisms to address these issues, particularly for women at risk of GBV and for adolescents and children with specific protection needs.

*People start drinking a lot, not all of them, but we’ve seen that and that leads to violence…within the family, towards women and children... Sometimes they are afraid or they don’t want everyone to know. They are ashamed. Or we can see the children, one day they are screaming and the next day they are silent and don’t talk at all. (INGO staff, Greek islands)*

While referral mechanisms were eventually implemented in the camps and AAFs, they were not always perceived to be effective according to stakeholders, to meet the psychosocial needs of refugees. Stakeholders reported that psychosocial services were limited in number and quality, and that there was a lack of coordination and communication between service providers.

### Challenges in healthcare delivery and meeting the needs of refugees

Healthcare providers reported many barriers to providing quality healthcare for the refugees in Greece, both prior to, and following the EU-Turkey agreement. Language and gender differences between providers and refugees were frequently reported as substantial barriers to care. Healthcare and social work providers that spoke Arabic or who were from the Middle Eastern region faced fewer challenges than providers working with interpreters and were better able to provide psychosocial support for their patients:

*I was a refugee, my family [was] the same. I can give them what they need. I feel exactly what they feel. I speak the Arabic language that most of them speak, the culture, the religion… [I understand] the ways that they are thinking. I find ways, strategies, or ways of psychological support to give them. (Social worker, Greek islands)*

Many stakeholders highlighted the ongoing need for trained medical interpreters, especially with the increased numbers of Syrian refugees seeking specialty care in Greek public and private facilities. It was often indicated that access and quality of care for refugees seeking services in Greek facilities depended on an Arabic-speaking provider or medical interpreter, with some patients not receiving care due to the absence of interpretation services.

Several participants emphasized the difficulties in addressing sexual and reproductive health, family planning and GBV for male healthcare providers due to gender sensitivities. Infrastructure and limited access to private spaces in clinics were highlighted as barriers to providing quality care. Female healthcare providers reported fewer barriers when discussing these issues, as one participant explained:

*The first day when I [took] a trip to the clinic it was [a] disaster because I don’t exaggerate if I told you 100 women [came] with gynecology [infections]… the doctor before me was from [the] military in Greece and he is [a] man… they cannot [tell] him. None of them [came] and [complained] about their problem… I’m a female doctor, the women [came] like ants. (Healthcare provider, Greek mainland)*

Considerations of language and gender were particularly relevant as NGOs started the process of transitioning health service provision to the local health authorities. Some stakeholders expressed concern about the departure of NGOs and the transition to care within the Greek health system, fearing that the absence of NGO health services would be detrimental to refugee health. They felt that the refugee population needed to be informed of the services available within the Greek health system and the local care-seeking processes. As one participant reflected:

*During the emergency, we have tried to support the hospital and the authorities. But now, we have to go back to a system where it is not [NGOs] filling in the gaps. But we have to make sure the public and the system are supported. (INGO Staff, Greek islands)*

Some participants commented that cash distributions and the introduction of medical booklets and Greek social insurance numbers, known as AMKA, for refugees were important initiatives that could potentially address these gaps and improve access to health services for refugees within Greek facilities, especially for those with chronic health conditions. As one healthcare provider explained:

*[The] AMKA number, it is the password to go to the hospital direct…AMKA makes your life easier actually. Because they can prescribe easier the medicines. (Healthcare provider, Greek islands).*

Efforts to provide the AMKA to some refugees were seen as practical steps to prepare for the transition from NGO-provided health services to those available through the Greek health system, however they were not provided across all camp sites and many stakeholders indicated that the future of refugee healthcare remained uncertain.

## Discussion

This qualitative study provides stakeholders’ perspectives on how the health needs of the Syrian refugee population on the Greek islands and mainland evolved over the course of the humanitarian response in Greece from 2015 to 2017 and following changes in migration policy. The data reveal a substantial shift in health needs and health service provision, evolving from first-aid and treatment of acute infections into a more pronounced demand for chronic disease management, sexual and reproductive services, care for GBV survivors, and treatment of mental health disorders following border closures and a period of reduced migratory flow. These findings also demonstrate the complex challenges in providing quality care for this population, including language and gender barriers, and highlight opportunities to strengthen refugee healthcare as service provision transitions from NGOs to local health authorities in Greece [12].

The data on health needs are consistent with facility-based studies reporting that acute physical health issues, such as hypothermia, dehydration, and infections, were among the most commonly treated conditions for Syrian refugees upon arrival to Greece [18, 25–27]. Similarly, these studies also documented the mental health needs of refugees in Greece, [18, 26, 27] including the association of violence exposure and mental health disorders [18, 27]. Our study builds on this growing evidence base by providing perspectives from healthcare providers and stakeholders—important perspectives missing from previous research and that are needed to inform programs and policy.

Our findings underline the challenges that healthcare providers experienced in delivering quality care, as well as the importance of building trust and including gender concordant services and medical interpretation for a population facing a myriad of psychological risks. This is also supported by previous research demonstrating how communication challenges limited the ability to provide emotional support to refugees [25]. Other studies have emphasized the importance of cultural competency [28] in providing psychosocial care [29] and the value of medical interpretation for refugees [27, 30, 31], with a study describing the importance of “cultural mediators” in its health facilities [18]. In order to address the risks of violence noted in our study and in other studies of Syrian refugees [16], it is even more critical to offer integrated, multi-disciplinary, and human-centered care.

This study highlights the need for the healthcare landscape to adapt to the protracted and increasingly complex refugee health needs in this setting, especially following changes in migration policy. As seen in other refugee settings [32], improving the quality of care requires shifting from a focused provision of health services to a more holistic approach which encompasses psychiatric care and psychological counseling [29]. Furthermore, our data indicate a growing need to increase psychosocial training for healthcare providers [21] and to strengthen referral mechanisms for mental healthcare and GBV support services as health services transition to local authorities. Strategic communication and coordination is also needed between NGOs [33] and Greek health authorities [31] to facilitate the transition of health service provision to the Greek healthcare system and improve access and quality of care for refugees. The refugee population should be informed of locations and means to access to local health services, standards and processes in the Greek healthcare system, and availability of medical interpreters during service delivery. These recommendations may also be relevant in similar settings hosting Syrian refugees.

The findings highlight the importance of considering perspective and temporality when drawing conclusions about the humanitarian response in Greece. In addition to providing data on perceived health needs among refugees, this study provides perspectives of healthcare providers and their needs, which are often overlooked when investigating humanitarian responses. It also considers how stakeholders’ experiences providing health services to refugees evolved in relation to the implementation of the EU-Turkey agreement, whereas the majority of studies on refugee health tend to analyze and interpret facility-based patient data in isolation of migration policies. Arsenijevic et al. also interpreted their findings from health facilities in Greece in relation to the closed borders, noting that the EU’s approach to blocking entry aggravated the predicament of Syrian refugees and the authors encouraged a more open “reception approach” to mitigate some of the noted health concerns [18]. Our study supports that the predicament of Syrian refugees, in regard to health status, risks of violence, and risks of mental health disorders, was noted to substantially deteriorate with the border closures and restricted movement that resulted from the agreement. Our study also highlights the predicament of healthcare providers and demonstrates that challenges they faced to provide care, emphasizing the importance of ensuring that humanitarian workers are trained and prepared to manage complex health needs that arise in relation to policy changes. Future research on health needs should continue to incorporate multiple perspectives and be designed to consider the policy implications on health.

This study aimed to explore the perspectives of stakeholders, and as such, we are unable to investigate the extent to which their views correspond with those held by the refugees themselves in terms of their own perceived health needs and experiences of receiving care. While stakeholders’ perspectives highlight healthcare providers’ experiences in direct service provision, it is challenging to disentangle the extent to which changes in health needs can be attributed to an increase in reporting to healthcare providers compared to an increase in incidence. Furthermore, while we did not set out to explore the temporal dimensions of migration and the health implications from the perspective of the refugees, this is implicit through some of our participants’ accounts, and should be further explored through interviews with refugees themselves.

Other limitations to this study include the potential for social desirability and recall bias in the participants’ accounts. The participants may have provided perspectives they thought the interviewers were seeking to document, or may have struggled to recall the chronology of events, particularly those who were involved in the refugee response over a long period. The cross-sectional nature of the study also limits conclusions about how individual stakeholder perspectives changed over time. While participants were aware that the study aimed to focus on service provision to Syrian refugees, it is not possible to isolate their perspectives to only refugees from Syria nor is it possible to draw conclusions from our data about refugees from other countries. Interviews conducted on Skype may have elicited different responses than those conducted in person or may have limited the interpretation of non-verbal responses by the interviewer. The use of snowball sampling may also have led us to interview stakeholders who held similar views or experiences, although this strategy also enabled us to recruit participants beyond our own networks. Further strengths of the study include the high response rate among participants, and the range of their roles and experiences at different stages of the humanitarian response in Greece.

## Conclusion

In conclusion, this study found that stakeholders coordinating and providing health services to Syrian refugees in Greece observed a shift in refugees’ health needs from acute care to longer-term chronic conditions, including mental health disorders and increased risks of GBV, following the implementation of the EU-Turkey Agreement in March 2016. These findings demonstrate the need to address the challenges that healthcare providers experience in providing care for the Syrian refugee population in the context of changing migration policy. As the provision of care transitions from NGOs to Greek health authorities, human-centered services such as medical interpretation, and psychosocial services should be provided, female healthcare providers and interpreters should be integral to medical teams, and referral mechanisms should be strengthened to increase access to specialized care.

## Abbreviations

AAF: Alternative Accommodation Facility
EU: European Union
GBV: Gender-based violence
INGO: International non-governmental organization
NCD: Non-communicable diseases
NGO: Non-governmental organization
UNHCR: United Nations High Commissioner for Refugee
WAHA: Women and Health Alliance International

## References

[1] United Nations Development Programme, United Nations High Commissioner for Refugees. 3RP. Regional Refugee and Resilience Plan 2016-2017 in Response to the Syria Crisis. 2015. http://www.3rpsyriacrisis.org/wp-content/uploads/2015/12/3RP-Regional-Overview-2016-2017.pdf. Accessed 13 Jul 2017.

[2] United Nations Development Programme, United Nations High Commissioner for Refugees. 3RP Regional Strategic Overview 2017–18 in Response to the Syria Crisis. 2017. http://www.3rpsyriacrisis.org/wp-content/uploads/2016/12/3RP-Regional-Strategic-Overview-2017-18.pdf. Accessed 2 Nov 2017

[3] United Nations High Commissioner for Refugees. Syria regional refugee response: Inter-agency Information Sharing Portal. 2016. http://data.unhcr.org/syrianrefugees/regional.php. Accessed 2 Nov 2017

[4] United Nations High Commissioner for Refugees (2017). Refugees/migrants emergency response - Mediterranean - regional overview.

[5] United Nations High Commissioner for Refugees (2015). Greece data snapshot (26 Dec.).

[6] Regulation (EU) No 604/2013 of the European Parliament and of the council of 26 June 2013. Official journal of the European Union. http://eur-lex.europa.eu/legal-content/EN/LSU/?uri=CELEX:32013R0604. Accessed 11 Jan 2018.

[7] European Council. EU-Turkey statement, 18 march 2016. 2016. http://www.consilium.europa.eu/en/press/press-releases/2016/03/18/eu-turkey-statement/. Accessed 2 Nov 2017.

[8] Rygiel K, Baban F, Ilcan S (2016). The Syrian refugee crisis: the EU-Turkey ‘deal and temporary protection. Glob Soc Policy.

[9] European Commission (2016). EU-Turkey statement: questions and answers. Brussels.

[10] United Nations High Commissioner for Refugees. Regional Refugee and Migrant Response Plan for Europe January to December 2017. 2016. http://www.unhcr.org/partners/donors/589497d07/2017-regional-refugee-migrant-response-plan-europe-january-december-2017.html. Accessed 4 Nov 2017.

[11] United Nations High Commissioner for Refugees. Greece. Factsheet 1 January - 31 May 2016. 2016. https://data2.unhcr.org/en/documents/download/49602. Accessed 31 Oct 2016.

[12] National Health Operations Center (EKEPY). Health Working Group Meeting, 21/06/2017. 2017. https://data2.unhcr.org/en/documents/download/58793. Accessed 2 Oct 2017.

[13] United Nations High Commissioner for Refugees. Greece Fact Sheet / 1–30 September 2017. 2017. https://data2.unhcr.org/en/documents/download/60346. Accessed 10 Nov 2017.

[14] Doocy S, Lyles E, Hanquart B, Woodman M (2016). Prevalence, care-seeking, and health service utilization for non-communicable diseases among Syrian refugees and host communities in Lebanon. Confl Health..

[15] Strong J, Varady C, Chahda N, Doocy S, Burnham G (2015). Health status and health needs of older refugees from Syria in Lebanon. Confl Health..

[16] Reese Masterson A, Usta J, Gupta J, Ettinger AS (2014). Assessment of reproductive health and violence against women among displaced Syrians in Lebanon. BMC Womens Health.

[17] Harvey C, Garwood R, El-Masri R (2013). Changing gender roles among refugees in Lebanon.

[18] Arsenijević J, Schillberg E, Ponthieu A, Malvisi L, Ahmed WAE, Argenziano S (2017). A crisis of protection and safe passage: violence experienced by migrants/refugees travelling along the western Balkan corridor to northern Europe. Confl Health.

[19] Jefee-Bahloul H, Barkil-Oteo A, Pless-Mulloli T, Fouad FM (2015). Mental health in the Syrian crisis: beyond immediate relief. Lancet.

[20] United Nations High Commissioner for Refugees. Initial assessment report: protection risks for women and girls in the European refugee and migrant crisis. 2015. http://www.unhcr.org/protection/operations/569f8f419/initial-assessment-report-protection-risks-women-girls-european-refugee.html. Accessed 13 Jul 2017.

[21] Priebe S, Giacco D, El-Nagib R (2016). Public health aspects of mental health among migrants and refugees: a review of the evidence on mental health Care for Refugees, asylum seekers and irregular migrants in the WHO European region. Helath Evidence Network Synthesis Report.

[22] Daynes L. The health impacts of the refugee crisis: a medical charity perspective. Clin Med (Northfield Il). 2016;16:437–40. 10.7861/clinmedicine.16-5-437 PMC6297302 27697805

[23] Pavli A, Maltezou H (2017). Health problems of newly arrived migrants and refugees in Europe. Journal of Travel Medicine.

[24] Pope C, Ziebland S, Mays N (2000). Qualitative research in health care. Analysing qualitative data. BMJ.

[25] Kousoulis AA, Ioakeim-Ioannidou M, Economopoulos KP (2016). Access to health for refugees in Greece: lessons in inequalities. Int J Equity Health.

[26] Hermans MPJ, Kooistra J, Cannegieter SC, Rosendaal FR, Mook-Kanamori DO, Nemeth B (2017). Healthcare and disease burden among refugees in long-stay refugee camps at lesbos. Greece Eur J Epidemiol.

[27] Shortall CK, Glazik R, Sornum A, Pritchard C (2017). On the ferries: the unmet health care needs of transiting refugees in Greece. Int Health.

[28] Grove N, Zwi AB (2006). Our health and theirs: forced migration, othering, and public health. Social Science & Medecine.

[29] Hassan G, Ventevogel P, Jefee-Bahloul H, Barkil-Oteo A, Kirmayer LJ (2016). Mental health and psychosocial wellbeing of Syrians affected by armed conflict. Epidemiol Psychiatr Sci.

[30] Hunter-Adams H-A, Hunter-Adams J (2017). A qualitative study of language barriers between south African health care providers and cross-border migrants. BMC Health Serv Res.

[31] Ekmekci PE. Syrian refugees, health and migration legislation in Turkey. J Immigr Minor Health. 2016:1–8. 10.1007/s10903-016-0405-3. 10.1007/s10903-016-0405-3 PMC5028239 26995181

[32] Rowley EA, Burnham GM, Drabe RM (2006). Protracted refugee situations: parallel health systems and planning for the integration of services. J Refug Stud.

[33] Kentikelenis AE, Shriwise A (2016). International organizations and migrant health in Europe. Public Health Rev.

